# Response to: can one teaspoon of trehalose a day mitigate metabolic syndrome and diabetes risks?

**DOI:** 10.1186/s12937-021-00687-4

**Published:** 2021-03-26

**Authors:** Chiyo Yoshizane, Akiko Mizote, Chikako Arai, Norie Arai, Rieko Ogawa, Shin Endo, Hitoshi Mitsuzumi, Shimpei Ushio

**Affiliations:** Hayashibara Co. Ltd., 675 Fujisaki, Naka-ku, Okayama, 702-8006 Japan

February 22, 2021

Dear Editor,

Thank you for contacting us regarding the comments and questions from Dr. Fred Brouns’ group about our paper “Daily consumption of one teaspoon of trehalose can help maintain glucose homeostasis: a double-blind, randomized controlled trial conducted in healthy volunteers” [1]. We appreciate their interest in our work and comments. What we would like to stress first is that we believe trehalose’s action of maintaining glucose tolerance occurs before it is degraded to glucose in the gut.

As Dr. Fred Brouns’ group mentioned the mechanism of action of trehalose was not discussed or provided in our publication. This paper was simply intended to report on the results of the use of trehalose in postprandial hyperglycemic human volunteers as published. Other studies that have been published have reported that trehalose may have physiologic activities apart from simply being a disaccharide that provides the two glucose molecules from which it is formed. Below are some examples suggesting that such independent mechanisms exist.

In a mouse study using a high fat diet, trehalose administration showed improvement of insulin resistance in comparison with maltose, glucose or fructose [2].

Furthermore, trehalose administration showed improvement of glucose tolerance, compared to a water control group [3]. From the above results, it is thought that trehalose has a unique function as compared to other saccharides.

As Drs. Brouns and Blaak pointed out, we also agree that the intake of 3.3g trehalose is not large enough to increase blood glucose levels. Therefore we recognize that the mechanism of the improvement effect of trehalose on glucose tolerance is not simply related to the lowering of blood glucose. Interestingly, in animal tests, the improvement effect on glucose tolerance was identified in an amount of trehalose that is equal to 1.2-10g per 60kg of human body weight [2, 3].

On the other hand, according to the literature regarding the effects of trehalose on saccharometabolism, such as improvement of glucose tolerance, there is some evidence to show a mechanism of how trehalose works in the gut.

Recently, our group reported that trehalose administration suppressed adipocyte hypertrophy for both the trehalase KO mouse [4] and the WT mouse models consuming a high fat diet [5]. This result suggests the possibility that trehalose may be related to this suppressive effect before it is degraded by trehalase into glucoses. From the test results, it is assumed that trehalose administration may suppress the development of metabolic syndrome by inhibiting the exponential growth of chylomicrons due to containment of lipid droplets in the gut epithelium. This occurs because the proportion of CLD (cytoplasmic lipid droplets) in jejunal epithelium was increased by consumption of trehalose, and there is a negative-correlation between jejunal epithelium containing lipid droplets and the size of mesenteric adipocytes. According to the paper by Murotomi et al., it was reported that trehalose administration showed a significant beneficial effect on the glucose tolerance of the Type II diabetes TSOD model mouse, and the suppression of NASH to inhibit the deposition of iron in duodenum as well [6]. Furthermore, there is a report to show that intake of trehalose increased regulatory T cells in the small intestine and improved hyperglycemia in a Type I diabetes model mouse [7].

From review of the above publications it would strongly suggest that trehalose, but not glucose has the ability to control factors that relate to adipocyte or insulin resistance of the pancreas through an effect on the enteral environment. While these are still hypothetical owing to the few human clinical studies on trehalose administration, it is believed that a mechanism for the physiological actions of trehalose, outside of the two glucose molecules from which it is formed, will be elucidated in the not too distant future.

The reason sucrose, not maltose, was chosen as the control in this clinical experiment was to design the protocol to reflect practical food applications. We have also conducted other animal studies in which maltose was used as a control; however, since sucrose is commonly used as a food ingredient, not maltose, it was decided to use sucrose.

Finally, regarding the concern about this study being conducted by using Hayashibara’s employees as test subjects, as we also mentioned in the paper, the test was conducted as a double-blind, parallel-group comparison study to exclude any bias as much as possible. Furthermore, this test was approved by an ethics committee that consisted of members from a third party organization. However, outsourcing organizations should be used to conduct future tests to avoid this concern.

We hope that this response addresses some of the comments and questions from Drs. Brouns and Blaak.

Sincerely yours,

Yoshizane Chiyo

HAYASHIBARA CO., LTD.

R&D Division
